# Increase of the FGFR1 signaling in the FGFR1-5-HT1A heteroreceptor complex in midbrain raphe 5-HT neuron systems via allosteric receptor-receptor interaction

**DOI:** 10.1186/2193-1801-4-S1-P4

**Published:** 2015-06-12

**Authors:** Dasiel O Borroto-Escuela, Manuel Narvaez, Antonio Jiménez-Beristain, Julia Oflijan, Luca Pinton, Michael Di Palma, Alexander O Tarakanov, Giuseppa Mudó, Luigi F Agnati, Natale Belluardo, Kjell Fuxe

**Affiliations:** Department of Neuroscience, Karolinska Institutet, Sweden; Department of Physiology, School of Medicine, University of Málaga, Spain; Russian Academy of Sciences, St. Petersburg Institute for Informatics and Automation, Russian Federation; Department of Experimental Biomedicine and Clinical Neurosciences, Laboratory of Molecular Neurobiology, University of Palermo, Italy

**Keywords:** fibroblast growth factor receptor, serotonin 5-HT1A receptor, heteroreceptor complexes

The ascending midbrain 5-HT neurons known to contain 5-HT1A autoreceptors may be dysregulated in depression due to a reduced trophic support. New findings show existence of FGFR1-5-HT1A heteroreceptor complexes in the rat hippocampus with a partial characterization of their interface and in midbrain raphe 5-HT nerve cells. With *in situ* Proximity Ligation Assay (PLA) and supported by co-location of the FGFR1 and 5-HT1A immunoreactivities in midbrain raphe 5-HT cells, evidence for the existence of FGFR1-5-HT1A heteroreceptor complexes were obtained in the dorsal and median raphe nuclei of the Sprague–Dawley rat. Their existence in the rat medullary raphe RN33B cell cultures was also established. After combined FGF-2 and 8-OH-DPAT treatment, a marked and significant increase in PLA positive clusters was found in the RN33B cells. Synergistic receptor-receptor interactions in these receptor complexes indicated their enhancing role in hippocampal plasticity. The existence of FGFR1-5-HT1A heteroreceptor complexes also in midbrain raphe 5-HT nerve cells open up the possibility that antidepressant drugs by increasing extracellular 5-HT levels, can cause an activation of the FGF-2/FGFR1 mechanism in these nerve cells as well. Therefore, the agonist modulation of the FGFR1-5-HT1A heteroreceptor complexes and their specific role is now determined in rat medullary raphe RN33B cells and in the caudal midline raphe area of the midbrain rich in 5-HT nerve cells. The combined icv treatment with FGF-2 and the 5-HT1A agonist 8-OHDPAT synergistically increased FGFR1 and ERK1/2 phosphorylation in the raphe midline area of the midbrain and in the RN33B cells. Cotreatment with FGF2 and the 5-HT1A agonist induced RN33B cell differentiation as seen from development of the increased number and length of extensions per cell and their increased 5-HT immunoreactivity. These signaling and differentiation events were dependent on the receptor interface since they were blocked by incubation with TMV but not by TMII of the 5-HT1A receptor. Taken together, the 5-HT1A autoreceptors by being part of a FGFR1-5-HT1A heteroreceptor complex in the midbrain raphe 5-HT nerve cells appears to have also a trophic role in the central 5-HT neuron systems besides playing a key role in reducing the firing of these neurons.

